# Bibliography

**Published:** 1901-03

**Authors:** 


### Bibliography.

---

Dental Anatomy Note-Book. For use in conjunction with Tomes's Dental Anatomy, the South Kensington Museum, and Personal Instruction. By Douglas Gabell, M.R.C.S., L.R.C.P., L.D.S. Claudius Ash & Sons, Limited, London, 1900.

This is a somewhat peculiar size note-book for students, being eleven by six inches. The author says of it, "In spite of complaints as to its unwieldiness, I have kept to the original size, because I wish the book to remain essentially a 'Note-Book,' the blank pages and the spaces in the text being left for diagrams and notes to be made by the student when reading up the subject of dental anatomy or being coached." The size of the book seems to the writer a special advantage, being convenient for memoranda.

This book is not of similar character to the note-books so common in American medical and dental colleges, for these are ordinarily imperfect synopses of the lectures, but is really a guide to the student, enabling him to select the salient points in any given subject, thus saving a waste of time in wandering through a maze of technicalities and verbose phraseology.

It is presumed that the subject-matter is mainly based upon the methods of teaching peculiar to the English dental curricula. More attention is there paid to comparative anatomy than in American dental colleges, and hence very much of this book would not be available to the American student under present arrangement of courses. This guide would seem, therefore, to be best adapted to the course of study in English schools.

"The first part is practically Tomes's Dental Anatomy condensed, the second is intended as a guide to the study of cases in the Central Hall of the South Kensington Museum, and the third is compiled from various sources."

X-LIGHT APPARATUS. For Physicians in the Country and Others. By William Rollins. Read before the Röntgen Society of the United States, December, 1900.

This valuable monograph deserves a more permanent setting than the author has given it. It is upon a subject but little understood, but one that interests, or should interest, dentists even more than physicians, for intelligent dentists are beginning to understand that the X-ray is an important adjunct in all serious regulating cases, and that without it the position of impacted teeth is practically impossible to determine.

The readers of the INTERNATIONAL DENTAL JOURNAL need not to be informed of Dr. Rollins's work in this and other lines of investigation. The pages of this journal have repeatedly given evidence of his original powers in this direction, as well as the introduction of many valuable appliances.

The work is profusely illustrated by very perfectly prepared engravings. These are necessary to explain the text, and even with them the novice may find it difficult to understand all the details of machinery requisite to produce the results. Dr. Rollins has aimed to produce these in the simplest manner consistent with commercial demands and a minimum amount of study from those for whom they are intended.

It is impossible to give all the author's methods used to overcome difficulties, but the following quotation will give his ideas suited for cities where the street current is available.

"The time will come when every physician will use X-light in diagnosis and treatment. At present the number employing it is very limited. One reason is the defective character of most of the apparatus commercially obtainable.

"In cities where the street current is available, it is practical to obtain from several firms coils and static machines to order, which by suitable changes can be made useful. What some of these changes are will be mentioned later. For physicians in the country, remote from electricity or water-power, the problem is a difficult one. This problem I have attempted to solve in several ways. . . . The cheapest arrangement consists of a twelve-inch Ritchie coil. . . . The coil is excited from four storage cells, which are kept constantly charged by twenty-four gravity cells. The apparatus furnishes enough current to light an X-light tube for an hour a day, using forty-three watts in the primary and employing the hammer break usually furnished with the coil. Of course, great attention must be paid to having the resistance of the tube such that this small amount of energy will be expended with the least waste. The plant requires no care except to renew the gravity cells at the end of from three to six months, according to the amount of time the light is used. The whole apparatus is enclosed in a case about the height and length of an ordinary book-case, the coil being on top. I used an apparatus of this kind for several weeks to test its value."

In the country where electricity or water are not available for power, "The gasoline engine rated at one horse-power by the makers develops sufficient power to run the static machine three hundred revolutions for one hour on a quart of gasoline."

It is impossible to follow the author through his thorough and generally technical explanation of his various efforts to perfect and simplify the machines, but that he has added materially to the means that will tend to make the use of the X-ray more general must be conceded, and could this be presented in book form it would be of value to those contemplating efforts in this direction. It seems to the writer that all dental colleges should not consider the list of apparatus complete without an X-ray machine. The necessity for this is more and more apparent through the multiplication of difficult cases constantly presenting in the clinics.

